# Immune Sensing of Lipopolysaccharide in Plants and Animals: Same but Different

**DOI:** 10.1371/journal.ppat.1005596

**Published:** 2016-06-09

**Authors:** Stefanie Ranf

**Affiliations:** Phytopathology, TUM School of Life Sciences Weihenstephan, Technical University of Munich, Freising-Weihenstephan, Germany; THE SAINSBURY LABORATORY, UNITED KINGDOM

Defense of attempted infection depends on the host’s ability to sense invading pathogens and rapidly activate immune responses. Pathogens, in turn, use a repertoire of evasion strategies and virulence factors to circumvent the host’s surveillance and defense systems. Lipopolysaccharide (LPS), a complex glycolipid covering the cell surface of gram-negative bacteria, is a virulence factor shielding bacteria from adverse host environments and is sensed by animal as well as plant immune receptors [1,2].

## LPS Structure and Function

**Fig 1:**
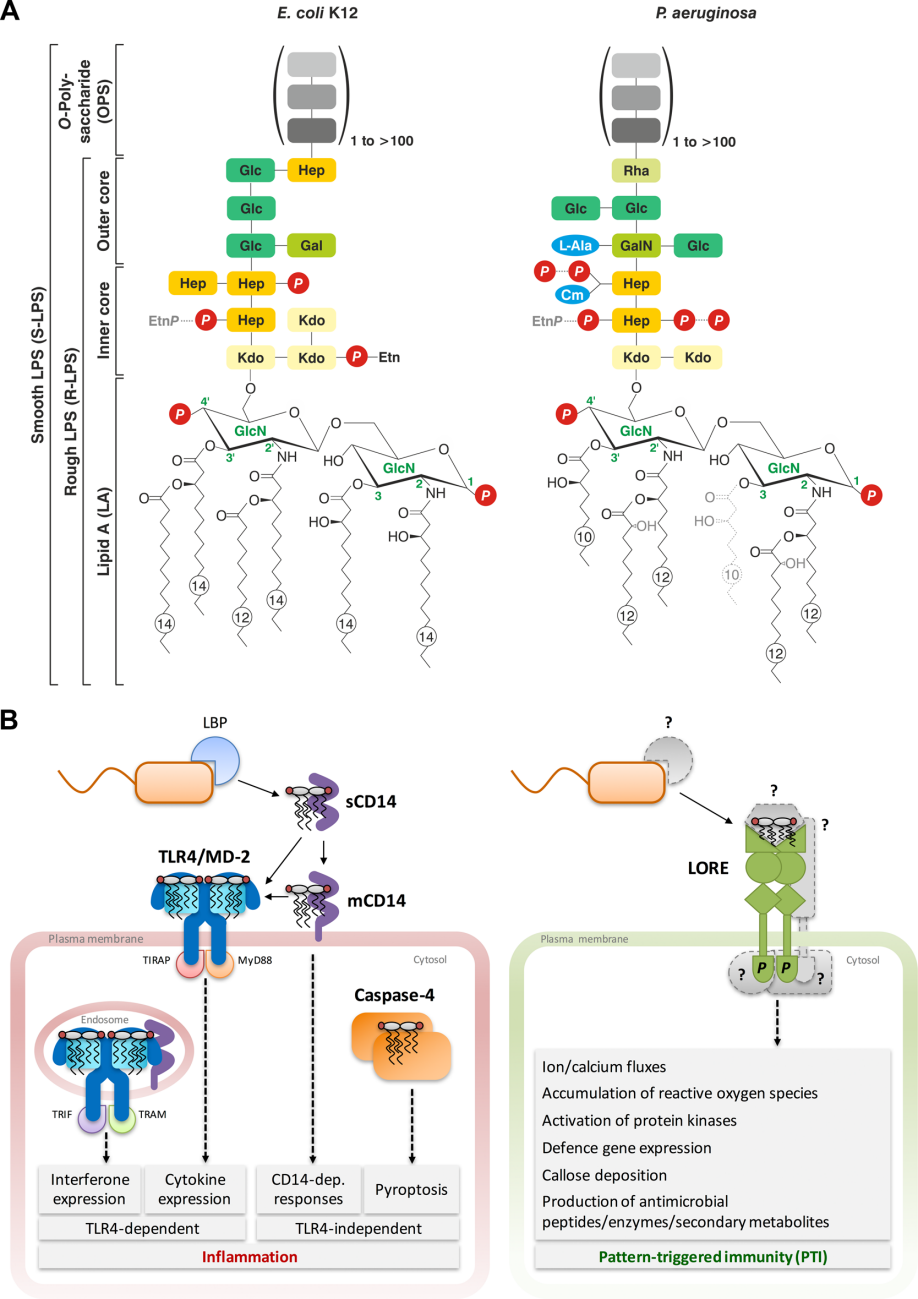
**Scheme of LPS structures of gram-negative bacteria (A) and the currently known LPS sensing systems in humans and cruciferous plants (B). A.** LPS structure showing a representative core region and LA from *Escherichia coli* K12 and *Pseudomonas aeruginosa*. The typical enterobacterial LA consists of a di-phosphorylated di-glucosamine with four primary and two secondary FAs (all C12/14) attached in an asymmetric fashion [3]. *Pseudomonas* spp. mostly produce penta-acylated and symmetrically hexa-acylated LA with shorter fatty acids (C10/12) [36]. The FAs are embedded in the OM, and the di-glucosamine is linked to the core oligosaccharide composed of about ten monosaccharides, which is conceptually subdivided into the rather variable outer core and the more conserved inner core region that usually contains heptose and the LPS-specific monosaccharide “Kdo” [3,36]. Often, an OPS consisting of repetitive units composed of several monosaccharides is attached to the core region. Dotted/grey lines indicate non-stoichiometric substitutions. Structures according to [36,37]. Abbreviations: Ara4N, 4-amino-4-deoxy-L-arabinose; Cm, carbamoyl; Etn, ethanolamine; Gal, galactose; GalN, galactosamine; Glc, glucose; GlcN, glucosamine; Hep, L-*glycero*-D-*manno*-heptose; Kdo, 3-deoxy-D-*manno*-oct-2-ulosonic acid; *P*, phosphate; Rha, L-rhamnose. **B.** In humans (left panel), LPS is sensed by different immune cells through different extra- and intracellular receptors [14]. LPS is disaggregated from the bacterial membrane by the serum protein LBP and transferred to CD14, which occurs as a soluble (sCD14) and membrane-linked (mCD14) version. Dependent on the cell type, CD14 can trigger LPS signaling itself, such as calcium signaling and activation of NFAT transcription factors in dendritic cells, or further transfers LPS to the membrane-resident TLR4/MD-2 receptor complex. LA binding to a preformed TLR4/MD-2 hetero-dimer leads to association with another TLR4/MD-2-dimer and initiates intracellular signaling. Depending on the cellular localization (at the plasma membrane or in endosomes upon CD14-dependent endocytosis), TLR4/MD-2/LPS complexes activate production of either interferons or cytokines through distinct signaling adapters (TIRAP/MyD88 or TRIF/TRAM) [14]. Intracellular LPS leads to oligomerization of caspase-4, activation of the non-canonical inflammasome and pyroptotic cell death [19]. In plants (right panel), the bulb-type lectin S-domain-1 RLK LORE (LipoOligosaccharide-specific Reduced Elicitation) was identified as the first LPS receptor component in plants and mediates sensitive perception of *Pseudomonas* LA [30]. If and how LPS is processed in the apoplast to make the membrane-embedded LA accessible for receptor binding, if LA directly binds to LORE or to an LPS-binding co-receptor and how the receptor complex and downstream signaling is activated, is yet unknown. In analogy to other SD-RLKs, LORE presumably forms dimers and is activated through mutual phosphorylation by the cytosolic kinase domain [38]. Taken together, both mammals and plants sense LA as pathogen-associated molecular pattern (PAMP) but with distinct epitope specificities and through different types of receptors.

LPS consists of three functionally distinct domains (Fig 1A) [3]: the lipophilic lipid A (LA), a di-glucosamine carrying four to seven fatty acids (FAs), is linked to an oligosaccharide core region that mostly carries an *O*-polysaccharide (OPS) consisting of a variable number of oligosaccharide repeats. OPS composition is highly diverse among bacterial species and strains. The FA pattern on the LA, as well as phosphorylation and other modifications on the LA, core oligosaccharide, or OPS, can also differ considerably. Thus, LPS structures vary substantially between bacterial species, likely due to adaptation to different environments and lifestyles, but also a single bacterial cell envelope comprises a mixture of different LPS variants with remarkable size and structural heterogeneity (Fig 1A) [2,3]. The primary stability and barrier functions of LPS are conferred by the rather conserved inner core-LA region [2]. Cross-linking of negative residues of the inner core and LA backbone through divalent cations (Mg^2+^/Ca^2+^) facilitates tight LPS packing, which is fundamental to bacterial outer membrane (OM) rigidity and low permeability (Fig 1A) [3]. The OPS chains are involved in adhesion processes and protect bacteria from hostile environments, e.g., host antibacterial agents, thereby promoting virulence [3]. Bacteria lacking OPS or negative core charges are generally non-virulent and cannot survive within animal or plant hosts [2,4].

## Perception of Molecular Patterns—A Common Concept of Animal and Plant Innate Immunity

Both animals and plants recognize evolutionary conserved pathogen-associated (PAMPs) and host-derived damage-associated molecular patterns (DAMPs) through germline-encoded pattern recognition receptors (PRRs) [1,5–9]. Bacterial cell surface components such as LPS, peptidoglycan, and flagellin are typical PAMPs, as they are vital for microbial survival and common to whole microbial classes [6–8].

In mammals, PAMPs are sensed by different classes of PRRs, e.g., the Toll-like receptors (TLRs), located on the cell surface, in endosomes, and the cytosol, and trigger inflammatory responses (Fig 1B) [5–7]. In addition to this innate immune system, vertebrates evolved an adaptive immune system employing highly specific antibodies produced through somatic gene rearrangements and clonal selection [6]. In plants, sensing of PAMPs and DAMPs by their respective PRRs induces a common set of signaling and defense responses known as pattern-triggered immunity (PTI) (Fig 1B) that results in local as well as systemic resistance [8,10,11]. All plant PRRs known to date reside on the cell surface and mostly are receptor-like proteins (RLPs) or receptor-like kinases (RLKs) [9]. These gene families have greatly expanded in plants compared to mammals, indicating a greater PAMP sensing capacity, possibly to compensate for the lack of adaptive immunity [10]. In contrast to mammals, no cytoplasmic microbial pathogens (except viruses) were observed in plants so far [12].

PAMP sensing is an integral part of animal and plant immunity. Downstream signaling and defense responses show conceptual similarities and share some molecular modules, but there are also substantial differences owing to the different organization of animals (specialized immune cells) and plants (nearly all cells capable of immune responses) [10–13]. Apparently, PAMP perception evolved independently in both kingdoms, as supported by sensing of distinct flagellin epitopes through different PRRs in plants and mammals [1,13].

## LPS Sensing in Mammals and Plants—Emerging Parallels but Distinct Specificities

In mammals, all LPS domains contribute to immune recognition (Table 1). OPSs trigger antibody production in the adaptive immune system which causes a selective pressure leading to extensive OPS diversification [2]. The LA domain is recognized in picomolar concentrations as PAMP by the innate immune system through different extra- and intracellular LPS sensors and induces inflammation (Fig 1B) [7,14]. Upon LPS binding, the TLR4/MD-2 (myeloid differentiation factor-2) complex triggers expression of pro-inflammatory mediators [14–17]. LPS binding to TLR4/MD-2 is facilitated by an LPS transfer cascade involving the serum protein LPS-binding protein (LBP), which extracts LA from the bacterial membrane, and the glycoprotein CD14 [18]. CD14 can also trigger LPS signaling independent of TLR4/MD-2 [14]. Cytosolic LPS/LA is further sensed through LPS-mediated activation of non-canonical inflammatory caspases (Fig 1B) [19]. Ultimately, LPS induces an array of defense responses such as activation of phagocytes and production of pro-inflammatory cytokines and interferones and antimicrobial peptides [6,7,14]. An exaggerated immune reaction to LA, also termed endotoxin, can result in sepsis and life-threatening septic shock [3,14]. In humans, enterobacterial LPS is the most potent activator of the TLR4/MD-2 and the caspase pathway, whereas *Pseudomonas* LPS is only a weak agonist because of structural differences within the LA (Fig 1A) [19–21].

**Table 1:** Prominent examples of functions of the different LPS domains in mammalian and plant immunity*.

	Mammals	Plants
**OPS**	Strongly antigenic in adaptive immunity; classification of bacterial strains according to serotypes [2,36]. Important for survival within the host and virulence [2,3,21].	Synthetic oligo-rhamnans (50–100 μg/ml) and *Burkholderia* OPS (50–100 μg/ml) induce defense gene expression in Arabidopsis [24,28]. Important for survival in plant tissue and virulence [4,29].
**Core region**	*P*.* aeruginosa* LPS is specifically internalized through its outer core oligosaccharide by cystic fibrosis transmembrane conductance regulator [39].	*Xanthomonas* core oligosaccharides (20–50 μg/ml) induce defense responses in Arabidopsis and tobacco [25,26,29].
**LA**	Typical enterobacterial LA is sensed as PAMP via TLR4/MD-2, CD14, and non-canonical inflammatory caspases and triggers inflammation [14,17,19]. Other LA structures (depending on the acylation and phosphorylation pattern and other modifications) are only weak agonists or even antagonists of TLR4/MD-2 signaling [3,17,20,33].	*Pseudomonas*/*Xanthomonas* LPS/LA (0.5–25 μg/ml) is sensed as PAMP via LORE in cruciferous plants and induces pattern-triggered immunity (PTI) [30]. LPS/LA (20–100 μg/ml) from diverse bacterial species (including *Pseudomonas*, *Xanthomonas*, *E*.* coli* and *Burkholderia cepacia*) induce defense responses in Arabidopsis, tobacco, and rice [22,23,26–29,31]. By contrast, [25] do not observe defense responses to *Xanthomonas* LA in tobacco.

*A comprehensive literature overview about plant LPS responses is available in [4,29]

LPS from different bacterial species also induces defense responses in various plant species, possibly through sensing of distinct epitopes by different perception systems (Table 1) [4,22–29]. The model plant *Arabidopsis thaliana*, for instance, responds with nitric oxide production, defense gene expression, and induced resistance to LPS from different bacteria, including *Burkholderia cepacia*, *Xanthomonas campestris*, *Pseudomonas* spp., and *Escherichia coli* [22,26,28,29]. Studies on plant LPS responses are difficult to compare (Table 1) [4,29], as different LPS preparations were used in different concentrations on diverse plant species. LPS cannot be synthesized but is extracted from complex bacterial cultures. This raised concerns about possible highly active contaminations in LPS preparations [1]. Isolated LPS might also cause unspecific stress to plants with increasing concentrations because of its amphiphilic nature. As LPS-sensing systems in plants remained genetically unidentified, these issues could not be undoubtedly resolved. Recently, however, it was found that the RLK LORE (LipoOligosaccharide-specific Reduced Elicitation), which belongs to the plant-specific class of bulb-type lectin S-domain-1 kinases (SD-RLKs), mediates sensitive perception of *Pseudomonas* and *Xanthomonas* LPS as PAMP in the model plant *A*. *thaliana* and other crucifers triggering typical PTI responses [30]. LORE loss-of-function mutant plants are insensitive to *Pseudomonas* and *Xanthomonas* LPS and hypersusceptible to *P*. *syringae* infection. Transient expression of LORE in LPS-insensitive tobacco plants confers LPS sensitivity, demonstrating a key function for LORE in sensing LPS. Chemical isolation of LA from *P*. *aeruginosa* LPS further proved that LA alone is sufficient to induce LORE-dependent PTI [30]. If LA directly binds to LORE, however, remains yet to be shown. Perception of the membrane-embedded LA might further require disaggregation from the bacterial membrane through unidentified LPS-binding plant proteins similar to those in mammals.

LORE specifically senses *Pseudomonas* and *Xanthomonas* but not the typical enterobacterial LPS, e.g., of *E*. *coli* [30]. Thus, both mammals and plants evolved to sense LPS via its LA domain, but, apparently, the detected epitopes are structurally distinct. Future studies will reveal which structural LA features determine the specificity of LORE-mediated LPS sensing in plants. Current data hint to an important role of the LA acylation pattern and phosphorylation [26,30,31]. As LORE does not detect enterobacterial or *B*. *cepacia* LPS, previously observed responses of *A*. *thaliana* to LPS from these species [22,28,29] cannot be mediated via LORE. It will be interesting to see, in the future, if other plant LPS immune sensors also target LA or other LPS epitopes and if they show a similar LPS recognition specificity as LORE.

Interestingly, plants possess several of the canonical LPS biosynthesis genes and produce LPS precursors resembling enterobacterial LPS, but their function is yet elusive [32]. Nevertheless, this might indicate a necessity for plants to evolve perception of a different, truly microbe-specific, LPS substructure to avoid auto-immunity against endogenous LPS-like compounds. Despite conceptual analogies, LA sensing with distinct epitope specificities in animals and plants through structurally unrelated receptors [14,17,19,30] further substantiates that plant and animal PAMP sensing evolved independently but might converge on predestined PAMP targets.

## LPS Remodeling in Host Adaptation—A Common Bacterial Virulence Strategy?

Many bacterial pathogens employ LPS structure alterations, either constitutive or through dynamic modifications, to adapt to changing environments and to avoid immune recognition [33]. According to different lifestyles, LPS modifications vary greatly between bacterial species but mostly affect the LA and inner core [33]. Negative LA/core charges are crucial for OM stability and LPS-TLR4/MD-2 interactions. They are targeted by host-derived cationic antimicrobial peptides (CAMPs), an ancient defense mechanism of vertebrates and plants, to interfere with LPS cross-linking [3,20,33]. Bacteria can mask negative LA/core charges by attaching cationic moieties resulting in increased CAMP resistance and impaired TLR4/MD-2 immune sensing [33]. Thus, plant-pathogenic bacteria may also deploy LPS modifications as a virulence strategy to enhance resistance to antibacterial host compounds and to interfere with immune detection. The common sensing of PAMPs such as flagellin or LPS by plants and animals allows pathogens to deploy similar virulence strategies to colonize animal as well as plant hosts [12,34].

## Conclusion

LPS has important functions in host–bacteria interactions, from shielding bacteria against adverse environments to host immune sensing [1,2], which are exerted by specific LPS part structures that can additionally be modified for adaptation to changing environments encountered during host colonization [20,33]. Studies on LPS structures and functions in plants, as well as the underlying genetic repertoire of plant-associated bacteria, will shed light on the evolutionary forces driving the recognition of the LA domain with distinct structural preferences as PAMP in animals and plants. On the practical side, understanding LPS immune sensing and LPS virulence functions of economically important plant pathogens, e.g., *Pseudomonas*, *Xanthomonas*, *Erwinia*, *Ralstonia*, or *Xylella* [35], will help to develop antibacterial tools and to improve disease resistance in crop plants in the future.
